# Exposure to Caffeinated Energy Drink Marketing and Educational Messages among Youth and Young Adults in Canada

**DOI:** 10.3390/ijerph16040642

**Published:** 2019-02-21

**Authors:** Danielle Wiggers, Mark Asbridge, N. Bruce Baskerville, Jessica L. Reid, David Hammond

**Affiliations:** 1 School of Public Health and Health Systems, University of Waterloo, Waterloo, ON N2L 3G1, Canada; dwiggers@uwaterloo.ca (D.W.); jl3reid@uwaterloo.ca (J.L.R.); 2 Department of Community Health and Epidemiology, Dalhousie University, Halifax, NS B3H 1V7, Canada; mark.asbridge@dal.ca; 3 Public Health Ontario, Toronto, ON M5G 1V2, Canada; bruce.baskerville@oahpp.ca; 4 School of Pharmacy, University of Waterloo, Kitchener, ON N2G 1C5, Canada

**Keywords:** energy drinks, marketing, educational messages, youth, young adults

## Abstract

The objective of the current study was to evaluate young Canadians’ exposure to caffeinated energy drink marketing and educational messages that warn about the potential health risks of energy drinks. An online survey was conducted in 2015 with youth and young adults aged 12–24 years recruited from a national online panel (*n* = 2023). Respondents were asked about their exposure to energy drink marketing and educational messages that warn about the potential health risks of energy drinks. Regression models were fitted to examine correlates of exposure to marketing and to educational messages. Over 80% of respondents reported ever seeing energy drink marketing through at least one channel, most commonly television (58.8%), posters or signs in a convenience or grocery store (48.5%), and online ads (45.7%). The mean number of marketing channels selected was 3.4 (SD = 2.9) out of ten. Respondents aged 18–19 (vs. 12–14 and 15–17) and 20–24 (vs. 12–14 and 15–17) reported significantly more channels of exposure to marketing. Overall, 32% of respondents reporting ever seeing an educational message about energy drinks. The most frequently reported sources of exposure were at school (16.2%), online (15.0%), and on television (12.6%). Respondents aged 18–19 (vs. 12–14, 15–17 and 20–24) and 20–24 (vs. 15–17) were significantly more likely to report having seen an educational message. Exposure to energy drink marketing was common among youth and young adults and was significantly more prevalent than exposure to educational messages that warn about the potential health risks of energy drinks. A comprehensive policy approach, including enforcing responsible marketing and increasing education surrounding the risks of consuming energy drinks, may be an effective approach in promoting lower-risk consumption of CEDs.

## 1. Introduction

With the increase in caffeinated energy drink (CED) marketing, there has also been an increase in consumption, which is concerning given the association of CED consumption with adverse health effects and other risky behaviors [1,2]. For example, previous related research showed that over half of youth and young adults in Canada who had ever used CEDs reported experiencing an adverse event following consumption, including fast heartbeat, sleeping difficulties, headaches, nausea/vomiting/diarrhea, chest pain, and seizures [1]. The Canadian adverse event data is consistent with US findings indicating substantial increases in CED-related emergency department visits between 2007 and 2011 [3]. Consumption of CEDs is also associated with other risky behaviors including alcohol use, smoking, and other substance use [2]. While caffeine is generally safe in quantities outlined by Health Canada, there appears to be greater risks from consuming CEDs in comparison to other sources of caffeine, like coffee [1]. 

Given the potential adverse health effects and risks, Health Canada does not recommend the consumption of CEDs by certain sub-populations, including children 12 years of age and younger [4]. For this reason, CEDs are prohibited from being marketed to children [4]. In addition, the Canadian Beverage Association has voluntary marketing codes restricting the advertisement of CEDs in programming (TV, radio, print, or digital) where 35% or more of the audience is under 12 years of age [5,6]. In addition to advertising to children, CEDs are also prohibited from being promoted for use during sports or with alcohol [4]. These marketing restrictions, along with other regulations such as cautionary “warning” statements on the product label, were implemented by Health Canada in 2012, and are part of the Temporary Marketing Authorization granted to CEDs [4].

To date, there is little evidence on the scope of CED marketing in Canada. In the US, CED marketing has risen dramatically in recent years. Advertising expenditures for energy drinks increased 2.5 times between the years 2008 to 2012 and beverage companies spent $175 million on advertising energy drinks in 2013 [7,8]. 

CED manufacturers typically target young people and males, reaching their target audience through a wide variety of marketing channels [8,9,10]. In Canada, previous related research on youth and young adults aged 12–24 found that over 80% of respondents reported ever seeing a CED advertisement, with TV being the most common source of exposure, followed by posters or signs in a convenience or grocery store, and ads on the internet [11]. Recent Canadian research showed that Red Bull Energy Drink is one of the top five most frequently advertised food and beverage products on children’s and teens’ favorite websites [12]. Further, qualitative research in the UK found that respondents aged 10–14 identified a wide variety of media through which they were targeted with CED promotional messages, including the internet, TV, computer games, bus stop advertisements, supermarket promotions, and sponsorship of sports or other events [13]. Indeed, CEDs are still commonly marketed to and consumed by young people and used within contexts that are advised against, such as during sports and with alcohol [11,14,15]. For example, Red Bull has a significant involvement with eSports, hosting events and sponsoring players, which has essentially allowed the brand to become part of the eSports market [16]. Research has also examined the impact of CED advertising. For example, one study found that exposure to digital marketing for the websites and social media sites of two popular brands of CEDs improved young adult participants’ attitudes, as well as purchase and consumption intention of energy drinks [17].

Given the widespread CED marketing and the potential health risks associated with energy drinks, public health authorities have called for increased public health measures surrounding CEDs, including increasing education regarding the health risks associated with consuming CEDs [9,18,19,20,21]. Public educational messages have been shown to be very important in some domains, including reducing smoking prevalence [22,23,24]; however, the impact of educational messages depends on the design and setting in which they are delivered [25,26]. To date, there is very little evidence on the scope of public education efforts aimed at CEDs or their impact on younger consumers. 

The current study sought to examine exposure to CED advertisements and promotions, as well as public education messages among Canadian youth and young adults. The study examined the prevalence of exposure across specific media and information channels, as well as associations with CED consumption and differences across sociodemographic groups. To our knowledge, the study is the first population-based study to examine exposure to public educational messages, and the “promotional” and “dissuasive” information environment for CED products among young people.

## 2. Materials and Methods

Data were collected via self-completed online surveys, between 6 November 2015 and 22 December 2015.

### 2.1. Sample and Recruitment

Respondents were recruited via email through the Legerweb consumer panel, which has over 400,000 active members, half of them sampled using probability-based methods [27]. Respondents aged 18–24 were recruited directly. Respondents aged 12–17 were recruited through their parents and parental consent was obtained prior to youth accessing the survey. All respondents were provided with information about the study and asked to give consent before participating. The survey was available in English or French and took approximately 20 min to complete. Respondents received remuneration from Léger in accordance with their usual incentive structure, which allows respondents to earn points or monetary rewards (redeemed as cash or donated), as well as chances to win monthly prizes.

A total of 2181 respondents completed the survey. Records were deleted due to missing data on variables used for weighting (*n* = 22) and other variables of primary interest (*n* = 32), completion outside of the study timeframe (*n* = 1), or failing a data quality check question that asked for the current month (*n* = 103). Thus, a total of 2023 responses were retained for analysis. Sample weights were constructed based on population estimates from the 2011 National Household Survey (NHS) [28]. Sample probabilities were created for 40 demographic groups (age group by sex by region) based on weighted NHS proportions, and applied to the data set. The study was reviewed by and received ethics clearance from the Office of Research Ethics at the University of Waterloo (ORE #19401). No personal identifiers were collected as part of the study.

### 2.2. Measures

#### 2.2.1. Sample Characteristics

Respondents were asked about the following sociodemographic characteristics: sex, age (categorized as 12–14, 15–17, 18–19, or 20–24), race/ethnicity (12 categories; re-coded as white (only), mixed/other/do not know/refused, or Aboriginal (any)), province of residence (recoded into region: British Columbia, Prairies, Ontario, Quebec, or Atlantic), mother’s education level (recoded as less than high school, high school graduate, college (some/completed), university (some/completed), or do not know/refused to answer), and usual grades in school. They were also asked if they had ever consumed energy drinks, if they watch or follow extreme sports, and if they had ever heard of mixing alcohol with energy drinks (see Table 1).

#### 2.2.2. Exposure to Energy Drink Marketing

Respondents were asked, “Have you ever seen the following types of ads or marketing for energy drinks?” and could select all that applied from the list of 10 channels shown in Table 2. A *Marketing Exposure Index,* ranging from 0–10, was created by summing the number of channels through which respondents reported they had seen CED marketing (refusals excluded; “do not know” recoded as “no”). For each channel selected, respondents were also asked about the last time they saw that type of marketing, with the following response options: *In the last 24 h; in the last 7 days; in the last 30 days; in the last 6 months; in the last 12 months; more than 12 months ago; do not know; refuse to answer*.

#### 2.2.3. Exposure to Educational Messages

Respondents were asked, “Have you seen or heard any educational messages that warn about the potential health risks of energy drinks? For example, in print, at school, on TV or radio, or other places.” and could select *yes; no; do not know;* or *refuse to answer.* If respondents selected “yes” they were asked, “Where have you seen educational messages that warn about the potential health risks of energy drinks?” and could select all that applied from the list of eight channels shown in Table 2. An *Educational Message Exposure Index*, ranging from 0–8, was created by summing the number of channels through which respondents reported they had seen an educational message (refusals excluded; “do not know” recoded as “no”). Respondents were also asked, “When was the last time you saw an educational message that warned about the potential health risks of energy drinks?” and could select one of the following response options: *In the last 24 h; in the last 7 days; in the last 30 days; in the last 6 months; in the last 12 months; more than 12 months ago; do not know; refuse to answer*. 

### 2.3. Analysis

Descriptive statistics were used to estimate prevalence of exposure to marketing and to educational messages that warn about the potential health risks of CEDs, overall, as well as by age group and sex. A negative binomial regression model was fitted to determine sociodemographic correlates of number of channels of exposure to energy drink marketing using the *Marketing Exposure Index.* Due to over dispersion, a negative binomial regression model was used, rather than a Poisson regression model. Using a count model is beneficial with count data because this type of model accounts for the use of non-negative integer values. A logistic regression model was fitted to determine sociodemographic correlates of any exposure to educational messages. Sex, age group, language, race/ethnicity, and region were included in each model. Additional covariates (maternal education, school grades, ever-use of energy drinks, extreme sports viewership/following, and awareness of alcohol mixed with energy drinks) were screened for inclusion in the models by testing bivariate correlations with the outcomes; those with an association at the *p* < 0.2 level were included in the model. The negative binomial regression model included all covariates. The logistic regression model included all covariates except school grades and ever-use of energy drinks. Reported estimates are weighted, unless otherwise specified. Analyses were conducted using IBM SPSS version 24 and SAS version 9.4.

## 3. Results

### 3.1. Sample Characteristics

Table 1 presents characteristics of the respondents in the analytic sample.

### 3.2. Exposure to Energy Drink Marketing

Table 2 presents channels where respondents reported seeing energy drink marketing, overall, as well as by age group and sex. Most respondents (81.8%) reported ever seeing marketing through at least one channel. Overall, the mean number of channels selected was 3.4 (SD = 2.9) out of the 10 listed. The most common sources of marketing exposure were ads on TV, posters or signs in a convenience or grocery store, and ads online/on the internet. The majority of respondents who reported seeing marketing had seen it within the last month. Among respondents who had seen ads on TV, 51.3% reported seeing one in the last 30 days, including 30.4% who reported seeing one in the last week. Among those who had seen posters or signs for CEDs in a convenience or grocery store, 76.3% reported seeing one in the last 30 days, including 40.5% seeing one in the last week. For those who had seen CED ads online/on the internet, 68.3% reported seeing one in the last 30 days, including 46.1% seeing one in the last week.

In a negative binomial regression model, the number of channels of exposure to CED marketing was significantly associated with age group, race/ethnicity, geographic region, ever-use of energy drinks, extreme sports viewership/following, and awareness of alcohol mixed with energy drinks (see Table 3). Older respondents, particularly those aged 18–19 (vs. 12–14 and 15–17) and 20–24 (vs. 12–14 and 15–17) reported a significantly greater number of channels of exposure to marketing. The difference between respondents aged 18–19 and 20–24 was of borderline significance, with those aged 18-19 reporting a greater number of channels. Respondents who were white (vs. mixed/other/not stated) reported a significantly greater number of channels of exposure to marketing. Although respondents who identified as Aboriginal reported the greatest number of channels of exposure, the difference was not significant compared to “white” respondents and was of borderline significance when compared to those who identified as mixed/other/not stated. This finding may be due to the smaller Aboriginal sample size. Respondents who resided in the Prairies reported a significantly greater number of channels of exposure to marketing, compared to British Columbia and Ontario. Those who reported they had ever consumed CEDs, watched or followed extreme sports, or had an awareness of mixing alcohol with energy drinks reported a significantly greater number of channels of exposure to marketing. No differences were observed for sex, language of survey, maternal education, or school grades.

### 3.3. Exposure to Educational Messages

In total, 32% (*n* = 647) of respondents reported they had seen an educational message. Table 2 presents channels where educational messages were seen, overall, as well as by age group and sex. Overall, the mean number of channels selected was 0.63 (SD = 2.9) out of 8. The most common sources of educational message exposure included at school, online/internet, and on TV. While most respondents (68%) had never seen an educational message, 1.1% saw an ad in the last 24 h, 2.3% saw one in the last seven days, 4.8% saw one in the last 30 days, 6.9% saw one in the last six months, 6.0% saw one in the last 12 months, 6.2% saw one more than 12 months ago, and 4.6% reported that they did not know the last time they saw one.

In a logistic regression model, reporting any exposure to educational messages was significantly associated with age group, language of survey, geographic region, maternal education, extreme sports viewership/following, and awareness of alcohol mixed with energy drinks (see Table 4). Respondents aged 18–19 (vs. 12–14, 15–17 and 20–24) and 20–24 (vs. 15–17) were significantly more likely to report having seen an educational message. Those who completed the survey in French were significantly more likely to report having seen an educational message than those who completed the survey in English. Residents of Quebec were significantly more likely to report having seen an educational message than residents of British Columbia and Ontario. Respondents whose mother completed high school were significantly more likely to report having seen an educational message than those who reported they did not know/not stated, and had a slightly higher likelihood than respondents whose mothers had less than a high school education. Those who watched extreme sports or had an awareness of mixing alcohol with energy drinks were also significantly more likely to report having seen an educational message. No differences were observed for sex or race/ethnicity.

## 4. Discussion

The current study suggests that over 80% of youth and young adults in Canada have seen marketing for energy drinks, many through multiple channels (an average of 3.4). Exposure to CED marketing has remained stable as the percentage of respondents who reported noticing any marketing was similar to the previous related study [11]. The most common sources of exposure were ads on TV, posters or signs in a convenience or grocery store, and ads online/on the internet, which is consistent with previous related research [11]. In addition, prevalence of exposure was high among many of the channels, consistent with previous findings indicating that young people are exposed to CED marketing through a wide variety of marketing channels [11,13]. 

Respondents who were older, had ever consumed energy drinks, watched or followed extreme sports, or who were aware of mixing alcohol with energy drinks were more likely to report a greater number of channels of exposure to CED marketing. These findings make sense given that marketing has consistently been shown to impact choices and behaviors [29,30,31,32] and so exposure to CED marketing would be expected to result in a greater likelihood of having consumed the product. In addition, those who have had experience and awareness of CEDs and CED-related attributes such as extreme sports and mixing alcohol with energy drinks would also be expected to have a greater exposure to CED marketing channels, given that marketing of CEDs is often featured alongside these attributes [8,16] and through multiple consumer targeted mediums [11,13]. Sex, language, maternal education, and school grades were not significantly associated with exposure to CED marketing channels. As CED marketing typically features content that is appealing to males [8,10], it was expected that males would have a greater exposure to CED marketing channels; however, the current findings suggest that CED marketing is reaching both males and females, aged 12–24 years, through a similar number of channels. This finding may be due to the broad range of marketing channels that are being used to reach consumers, some of which may have similar reach among both males and females.

To our knowledge, the current study is the first to examine exposure to educational messages that warn about the potential health risks of energy drinks. The results indicate that exposure to educational messages was relatively low in comparison to exposure to marketing, reported by only about one-third of respondents. Out of eight possible channels of educational message exposure, respondents reported an average exposure of less than one channel. The findings are not surprising given that we are unaware of any comprehensive campaigns that educate consumers of the health risks associated with consuming CEDs. In contrast to marketing, if there are public health education campaigns, they are less prominent or visible. To illustrate, in a qualitative study conducted with young people aged 12–25 in Australia, many respondents were oblivious to even the cautionary statements provided on energy drink cans, suggesting that visibility of such messages needs to be increased [33]. The respondents also suggested educational intervention strategies, among many other strategies, to reduce energy drink consumption, including implementing school visits and interactive educational sessions, having health messages show on news stories or television ads, educating parents, and developing practitioner-based strategies [33]. Previous research has also found that changing young people’s attitudes and perceived norms surrounding energy drinks may counter marketing effects on young people [34].

Given that educational message interventions generally produce the most effective behavior change outcomes when combined with other strategies [25,35], other components such as increasing price, restricting sales to minors, changing product packaging, enforcing responsible marketing, and reducing visibility in retail outlets may also be warranted [33]. Respondents who were older, completed the survey in French, resided in Quebec, were extreme sports viewers/followers, or had an awareness of mixing alcohol with energy drinks were more likely to report having seen an educational message. The association between speaking French and residing in Quebec and having a greater exposure to educational messages surrounding CEDs warrants further research, such as a survey of regional differences in the implementation of educational campaigns. Additional research is required to test and inform the content of educational messages for CEDs, which will help to assess the efficacy of educational initiatives.

### Limitations

The current study has limitations common to survey research. The sample was recruited through a web panel and, as such, was not probability-based, which may limit generalizability. Web panels pose issues such as self-selection bias as members opt-in. Further, nonresponse, either in recruitment (non-contact, refusal, or unavailability) or through attrition, is usually prevalent with web panels. However, the sample included all provinces and survey weights were applied to match national estimates for age, sex, and geographic region. Recall bias is also a possibility. For example, those who find certain ads relevant may be more likely to remember them, leading to selective recall. Nonetheless, the current findings are consistent with data showing that TV accounts for the majority of advertising expenditure for energy drinks [7], as well as findings from previous related research [11]. Lastly, the survey was completed in 2015 and it is possible that the results may differ if the survey were to be repeated in 2019. 

## 5. Conclusions

Findings from the current study indicate that exposure to CED marketing is prevalent among youth and young adults in Canada, significantly more so than exposure to educational messages that warn about the potential health risks of CEDs. Current regulations enacted by Health Canada, as well as the beverage industry’s voluntary marketing codes, prohibit the marketing of CEDs to children; however, this study, along with other previous studies, provides evidence that current policies are ineffective in this regard, and CED marketing is reaching young people [7,8,11,12,13]. Regulatory enforcement or amendments may help to address the ineffectiveness of current policies. In addition, while health professionals have called for an increase in education regarding the risks of energy drinks, the current study reiterates that exposure to educational messages is low. As educational messages are typically more effective when combined with other strategies, a comprehensive policy approach, as has been successful in reducing smoking prevalence, may be an effective approach in promoting lower-risk consumption of CEDs.

## Figures and Tables

**Table 1:** Sample characteristics of respondents in analytic sample, unweighted and weighted (*n* = 2023).

Characteristic	Unweighted % (*n*)	Weighted %
**Sex**		
Male	50.4% (1004)	51.2%
Female	49.6% (1019)	48.8%
**Age Group**		
12–14	19.5% (395)	21.3%
15–17	30.3% (612)	23.5%
18–19	10.4% (210)	15.9%
20–24	39.8% (806)	39.3%
**Language of Survey**		
English	60.3% (1220)	78.0%
French	39.7% (803)	22.0%
**Race/Ethnicity**		
White (only)	74.0% (1498)	67.3%
Mixed/Other/Not stated	22.9% (463)	28.9%
Aboriginal (any)	3.1% (62)	3.8%
**Region**		
British Columbia	7.4% (150)	12.9%
Prairies (AB, SK, MB)	13.3% (269)	18.5%
Ontario	30.8% (623)	39.5%
Quebec	43.2% (874)	22.5%
Atlantic (NB, NL, NS, PEI)	5.3% (107)	6.5%
**Maternal Education Level**		
Less than high school	7.3% (147)	7.8%
High school graduate	18.9% (382)	18.3%
College (some/completed)	31.9% (645)	31.2%
University (some/completed)	39.7% (804)	40.2%
Do not know/Not stated	2.2% (45)	2.5%
**School Grades**		
Below 50% (Mostly Fs)	0.3% (7)	0.3%
50–59% (Mostly Ds)	1.1% (23)	1.1%
60–69% (Mostly Cs)	9.9% (201)	10.0%
70–79% (Mostly Bs)	34.1% (690)	33.7%
80–89% (Mostly As or A+s)	41.3% (835)	41.6%
90–100% (Mostly A+)	11.5% (233)	11.3%
Do not know/Not stated	1.7% (34)	2.0%
**Ever-Use of Energy Drinks**		
Yes	74.2% (1501)	75.4%
No	25.8% (522)	24.6%
**Extreme Sports Viewer/Follower**		
Yes	15.8% (319)	15.3%
No	84.2% (1704)	84.7%
**Aware of Alcohol Mixed with Energy Drinks**		
Yes	69.1% (1397)	67.2%
No	30.9% (626)	32.8%

AB, Alberta; SK, Saskatchewan; MB, Manitoba; NB, New Brunswick; NL, Newfoundland; NS, Nova Scotia; PEI, Prince Edward Island.

**Table 2:** Channels where energy drink marketing and educational messages were ever seen, overall, as well as by age group and sex (*n* = 2023).

	Overall	Age 12–14	Age 15–17	Age 18–19	Age 20–24	Male	Female
**Channels Marketing Seen**							
Ads on TV	58.8% (1189)	54.2% (234)	52.0% (248)	68.0% (218)	61.6% (490)	62.0% (642)	55.4% (547)
Posters or signs in a convenience or grocery store	48.5% (982)	44.7% (193)	46.4% (221)	53.3% (171)	50.0% (397)	48.5% (502)	48.6% (479)
Ads online/on the internet	45.7% (924)	40.2% (173)	40.8% (194)	53.9% (173)	48.3% (384)	49.0% (508)	42.2% (417)
As part of social media sites, like Facebook or Twitter	39.9% (807)	28.7% (124)	31.6% (151)	49.6% (159)	46.9% (374)	40.1% (415)	39.7% (392)
Promotion or sponsorship, such as logos or links with events, sports teams, or athletes	37.8% (764)	26.7% (115)	32.0% (153)	50.1% (161)	42.2% (335)	39.4% (408)	36.1% (356)
Cars/vehicles with energy drink branding	34.1% (689)	26.8% (116)	22.3% (106)	43.9% (141)	41.1% (327)	36.4% (377)	31.6% (312)
Ads in magazines or newspapers	29.3% (592)	26.1% (112)	25.4% (121)	32.5% (104)	32.0% (255)	29.3% (304)	29.2% (288)
Free samples of energy drinks or shots	27.5% (557)	12.3% (53)	17.0% (81)	36.0% (115)	38.7% (308)	28.2% (293)	26.8% (264)
Giveaways of branded merchandise	20.9% (422)	13.1% (57)	10.4% (50)	29.5% (95)	27.8% (221)	22.2% (230)	19.5% (192)
Other	0.7% (15)	0.3% (1)	0.9% (4)	1.5% (5)	0.5% (4)	1.0% (10)	0.5% (4)
None of the above	13.1% (265)	16.3% (70)	15.9% (76)	7.3% (24)	12.0% (95)	12.5% (129)	13.7% (135)
Do not know	5.1% (103)	6.9% (30)	5.1% (25)	3.9% (13)	4.5% (36)	4.7% (48)	5.5% (54)
*Marketing Exposure Index* * (mean; SD)	3.43; 2.9	2.73; 2.6	2.79; 2.5	4.18; 3.0	3.89; 3.1	3.56; 3.0	3.29; 2.8
**Channels Educational Messages Seen**							
N/A—have not seen	68.0% (1376)	73.3% (316)	74.3% (354)	56.1% (180)	66.2% (526)	67.4% (698)	68.6% (677)
At school	16.2% (328)	17.2% (74)	13.9% (66)	27.2% (87)	12.6% (101)	17.7% (183)	14.7% (145)
Online/internet	15.0% (304)	9.7% (42)	12.2% (58)	22.8% (73)	16.4% (131)	14.6% (152)	15.4% (152)
On TV	12.6% (255)	12.0% (52)	11.2% (53)	12.5% (40)	13.8% (110)	13.4% (139)	11.8% (116)
Newspaper or magazine	6.7% (136)	4.8% (21)	5.2% (25)	6.5% (21)	8.8% (70)	6.2% (64)	7.3% (72)
Poster or billboard	4.6% (93)	3.9% (17)	3.2% (15)	5.5% (18)	5.5% (44)	4.9% (51)	4.3% (42)
On the radio	4.0% (81)	2.7% (12)	2.9% (14)	6.0% (19)	4.6% (36)	4.3% (45)	3.7% (36)
In a store	3.0% (62)	3.2% (14)	1.8% (8)	3.6% (12)	3.5% (28)	3.5% (36)	2.6% (25)
Somewhere else	0.7% (14)	1.1% (5)	0.9% (4)	0.2% (1)	0.6% (5)	0.4% (4)	1.0% (10)
Do not know	1.8% (36)	1.2% (5)	0.5% (2)	3.1% (10)	2.4% (19)	1.4% (15)	2.2% (22)
*Educational Message Exposure Index* ** (mean; SD)	0.63; 2.9	0.55; 1.2	0.51; 1.1	0.84; 1.4	0.66; 1.2	0.65; 1.2	0.61; 1.2

Note: Percentages do not sum to 100, as respondents could select multiple response options. * *Marketing Exposure Index* is the sum of the number of channels where marketing was seen (range 0–10); ** *Educational Message Exposure Index* is the sum of the number of channels where educational messages were seen (range 0–8).

**Table 3:** Results from a negative binomial regression model, showing correlates of reporting a greater number of marketing channels, as defined by the *Marketing Exposure Index* (0–10) (*n* = 2023).

Characteristic (mean; SD)	X^2^, *p*-Value	Incidence Rate Ratio (95% CI)	*p*-Value
**Sex**	1.33, *p* = 0.25		
**Age Group**	43.5, *p* < 0.001		
18–19 (4.2; 3.0) vs. 12–14 (2.7; 2.6)		1.4 (1.2, 1.6)	<0.001
18–19 (4.2; 3.0) vs. 15–17 (2.8; 2.5)		1.4 (1.2, 1.6)	<0.001
18–19 (4.2; 3.0) vs. 20–24 (3.9; 3.1)		1.1 (1.0, 1.2)	0.05
20–24 (3.9; 3.1) vs. 12–14 (2.7; 2.6)		1.2 (1.1, 1.4)	<0.001
20–24 (3.9; 3.1) vs. 15–17 (2.8; 2.5)		1.3 (1.1, 1.4)	<0.001
15–17 (2.8; 2.5) vs. 12–14 (2.7; 2.6)		1.0 (0.9, 1.1)	0.66
**Language of Survey**	1.13, *p* = 0.29		
**Race/Ethnicity**	7.93, *p* = 0.02		
Aboriginal (4.2; 3.0) vs. mixed/other/not stated (3.0; 2.8)		1.2 (1.0, 1.5)	0.06
Aboriginal (4.2; 3.0) vs. White (3.6; 2.9)		1.1 (0.9, 1.3)	0.43
White (3.6; 2.9) vs. mixed/other/not stated (3.0; 2.8)		1.1 (1.0, 1.2)	0.01
**Region**	9.56, *p* = 0.049		
Prairies (3.8; 2.9) vs. British Columbia (3.0; 2.9)		1.2 (1.0, 1.4)	0.02
Prairies (3.8; 2.9) vs. Ontario (3.3; 2.9)		1.1 (1.0, 1.3)	0.02
Prairies (3.8; 2.9) vs. Quebec (3.5; 2.8)		1.0 (0.9, 1.2)	0.65
Prairies (3.8; 2.9) vs. Atlantic (3.6; 2.7)		1.0 (0.8, 1.2)	0.75
**Maternal Education Level**	7.26, *p* = 0.12		
**School Grades**	8.82, *p* = 0.18		
**Ever-Use of Energy Drinks**	3.91, *p* = 0.048		
Yes (3.7; 2.9) vs. No (2.7; 2.7)		1.1 (1.0, 1.2)	0.048
**Extreme Sports Viewer/Follower**	21.94, *p* < 0.001		
Yes (4.0; 3.1) vs. No (3.3; 2.9)		1.3 (1.2, 1.4)	<0.001
**Aware of Alcohol Mixed with Energy Drinks**	167.43, *p* < 0.001		
Yes (4.1; 2.9) vs. No (2.1; 2.3)		1.8 (1.6, 1.9)	<0.001

**Table 4:** Results from a logistic regression model, showing correlates of having ever seen an educational message that warns about the potential health risks of energy drinks (*n* = 2023).

Characteristic (%)	X^2^, *p*-Value	Adjusted Odds Ratio (95% CI)	*p*-Value
**Sex**	0.01, *p* = 0.93		
**Age Group**	33.52, *p* < 0.001		
18–19 (43.9%) vs. 12–14 (26.7%)		2.2 (1.6, 3.1)	<0.001
18–19 (43.9%) vs. 15–17 (25.7%)		2.4 (1.8, 3.3)	<0.001
18–19 (43.9%) vs. 20–24 (33.8%)		1.7 (1.3, 2.2)	<0.001
20–24 (33.8%) vs. 15–17 (25.7%)		1.4 (1.1, 1.9)	0.009
20–24 (33.8%) vs. 12–14 (26.7%)		1.3 (1.0, 1.7)	0.07
15–17 (25.7%) vs. 12–14 (26.7%)		0.9 (0.7, 1.2)	0.53
**Language of Survey**	5.22, *p* = 0.02		
French (49.4%) vs. English (27.1%)		1.7 (1.1, 2.8)	0.02
**Race/Ethnicity**	4.29, *p* = 0.12		
**Region**	20.40, *p* < 0.001		
Quebec (48.7%) vs. British Columbia (21.1%)		2.1 (1.2, 3.6)	0.009
Quebec (48.7%) vs. Ontario (26.2%)		1.6 (1.1, 2.6)	0.047
Quebec (48.7%) vs. Prairies (28.1%)		1.6 (0.9, 2.6)	0.09
Quebec (48.7%) vs. Atlantic (42.8%)		0.8 (0.4, 1.4)	0.37
**Maternal Education Level**	13.15, *p* = 0.01		
High school (37.8%) vs. Do not know/not stated (12.7%)		4.3 (1.8, 10.3)	0.001
High school (37.8%) vs. Less than high school (28.2%)		1.5 (1.0, 2.3)	0.05
High school (37.8%) vs. College (30.8%)		1.3 (0.9, 1.7)	0.11
High school (37.8%) vs. University (32.3%)		1.2 (0.9, 1.6)	0.24
**Extreme Sports Viewer/Follower**	15.83, *p* < 0.001		
Yes (40.4%) vs. No (30.5%)		1.7 (1.3, 2.3)	<0.001
**Aware of Alcohol Mixed with Energy Drinks**	29.80, *p* < 0.001		
Yes (37.3%) vs. No (21.1%)		1.9 (1.5, 2.4)	<0.001
